# A patient with van Maldergem syndrome with endocrine abnormalities, hypogonadotropic hypogonadism, and breast aplasia/hypoplasia

**DOI:** 10.1186/s13633-017-0052-z

**Published:** 2017-10-13

**Authors:** Juan Sotos, Katherine Miller, Donald Corsmeier, Naomi Tokar, Benjamin Kelly, Vijay Nadella, Huachun Zhong, Amy Wetzel, Brent Adler, Chack-Yung Yu, Peter White

**Affiliations:** 1 Section of Endocrinology, Nationwide Children’s Hospital, 700 Children’s Drive, Columbus, OH 43205 USA; 2 Department of Pediatrics, College of Medicine, The Ohio State University, Columbus, OH 43210 USA; 3 Molecular & Human Genetics, The Research Institute at Nationwide Children’s Hospital, Columbus, OH 43205 USA; 4 The Institute for Genomic Medicine, Nationwide Children’s Hospital, 700 Children’s Drive, Columbus, OH 43205 USA; 5 Department of Radiology, Nationwide Children’s Hospital, Columbus, OH 43205 USA; 6 College of Medicine, The Ohio State University, Columbus, OH 43210 USA

**Keywords:** Breast aplasia, Breast hypoplasia, Congenital malformation, DCHS1, Hypogonadotropic hypogonadism, Intellectual disability, Osteopenia, Skeletal dysplasia, Van Maldergem Syndrome

## Abstract

**Background:**

We report a female patient with endocrine abnormalities, hypogonadotropic hypogonadism and amazia (breasts aplasia/hypoplasia but normal nipples and areolas) in a rare syndrome: Van Maldergem syndrome (VMS).

**Case presentation:**

Our patient was first evaluated at age 4 for intellectual disability, craniofacial features, and auditory malformations. At age 15, she presented with no breast development and other findings consistent with hypogonadotropic hypogonadism. At age 37, she underwent whole exome sequencing (WES) to identify pathogenic variants. WES revealed compound heterozygous variants in *DCHS1* (rs145099391:G > A, p.P197L & rs753548138:G > A, *p*.T2334 M) [RefSeq NM_003737.3], diagnostic of Van Maldergem syndrome (VMS-1). VMS is a rare autosomal disorder reported in only 13 patients, characterized by intellectual disability, typical craniofacial features, auditory malformations, hearing loss, skeletal and limb malformations, brain abnormalities with periventricular neuronal heterotopia and other variable anomalies. Our patient had similar phenotypic abnormalities. She also had hypogonadotropic hypogonadism and amazia. Based on the clinical findings reported, two previously published patients with VMS may also have been affected by hypogonadotropic hypogonadism, but endocrine abnormalities were not evaluated or mentioned.

**Conclusion:**

This case highlights an individual with VMS, characterized by compound heterozygous variants in *DCHS1*. Our observations may provide additional information on the phenotypic spectrum of VMS, including hypogonadotropic hypogonadism and amazia. However, the molecular genetic basis for endocrine anomalies observed in some VMS patients, including ours, remains unexplained.

**Electronic supplementary material:**

The online version of this article (10.1186/s13633-017-0052-z) contains supplementary material, which is available to authorized users.

## Background

Van Maldergem syndrome is a rare autosomal recessive disorder reported in only 13 patients (to our knowledge), characterized by intellectual disability, typical craniofacial features, auditory malformations, hearing loss, skeletal and limb malformations, brain abnormalities with periventricular neuronal heterotopia and other variable anomalies (Table 1). We reviewed the syndrome in Online Mendelian Inheritance in Man (OMIM) [1]; no endocrine abnormalities were listed in the features of VMS-1 (OMIM #601390) or VMS-2 (OMIM #615546), even though 2 previously reported patients probably had hypogonadotropic hypogonadism. VMS-1 is caused by recessive mutations in the *DCHS1* gene (dachsous cadherin-related 1 [Homo sapiens]) on chromosome 11p15.4, while VMS-2 is caused by recessive mutations in the *FAT4* gene (Fat tumor suppressor, Drosophila of 4), on chromosome 4q28.1.

**Table 1:** Characteristics of Van Maldergem Syndrome

Neurodevelopmental
Infantile hypotonia Developmental delay
Feeding difficulties early
Craniofacial features
Large forehead Flat face – maxillary hypoplasia Hypertelorism, Epicanthal folds Narrow palpebral fissures Pear shaped nose Dental malocclusion
Ear abnormalities
Small deformed ears Hearing loss
Upper airway obstruction
Choanal atresia/stenosis Tracheomalacia
Limb anomalies
Camptodactyly Palmar/plantar interphalangeal webbing Short metacarpals & phalanges Hyperlaxity of joints
Genitourinary
Hypospadias in one male Small kidneys
Radiological features
Sclerotic base of the skull Thickened frontal bone Maxillary hypoplasia Narrow thorax with short clavicles Limb bone abnormalities (metacarpals, phalanges, others) Abnormal pattern of ossification Scoliosis Generalized osteopenia
MRI brain findings
Thin optic nerves Abnormal corpus callosum Periventricular heterotopia Large lateral ventricles Reduced volume of pons & cerebellum

Features of VMS were originally described by Lionel Van Maldergem et al.*,* in 1992 [2]. Van Maldergem et al., described an 11-year-old girl with intellectual disability (IQ of about 50), peculiar facial appearance including a large forehead, broad nasal bridge, with broad bulbous nose (pear shaped nose), hypertelorism, bilateral epicanthus, narrow palpebral fissures, small ears and helix malformation, hearing loss, dental malocclusion, camptodactyly of the 3rd and 5th fingers, clinodactyly, short 5th metacarpals, genu recurvatum, and hyperlaxity of the major joints. Echocardiogram, chest x-rays, and EKG, were normal. Radiographs showed maxillary hypoplasia, and malformed external ears. The karyotype was 46, XX. Zampino et al. described a 5 year-old girl with a similar phenotype [3]. An MRI of her brain showed enlarged lateral ventricles, and poor outline of the infundibular recess of the 3rd ventricle, and the optic nerves were abnormally thin. Additionally, there was marked reduction of the thickness of the white matter, a thin corpus callosum, abnormally low inserted tentorium with reduced volume of the pons and hypoplastic superior vermis and cerebellar tonsils. They suggested the name of Cerebro-facial-articular syndrome.

In 2012, Mansour et al.*.* described 6 additional patients as well as a follow up of the original patient [4]. The skeletal dysplasia and anomalous neuronal migration (periventricular neuronal heterotopia) were also shown to constitute common, but variable components of this phenotype. Radiological features included osteopenia, and skeletal abnormalities. The original patient when she was 32 years old had no breast or pubertal development. In 2012, Neuhann et al. [5] described a 4-year-old boy from 1st cousin parents, with severe developmental delay, with similar craniofacial, dysmorphic features and skeletal abnormalities. He, in addition, had genital abnormalities (microphallus, bifid scrotum, and cryptorchidism) and hypoplastic mammilae. Capello et al. in 2013 [6] conducted studies in 9 individuals from 7 families, 5 previously reported and 2 of their own. In 4 patients, the authors identified new non-synonymous homozygous sequence variants in *DCHS1*: Van Maldergem syndrome 1.

*DCHS1* gene encodes a transmembrane calcium-dependent cell-cell adhesion molecule that belongs to the protocadherin superfamily, located on chromosome 11p15.4. Cell-cell adhesion is fundamental for multicellular architecture. Several classes of adhesion molecules are used to achieve this and cadherins represent a major family of such molecules. DCHS1 protein is the ligand for the *FAT4* receptor. Additional human studies in 5 patients by Capello et al. [6] from 4 unrelated families (2 siblings in one family) led to identification of biallelic missense and nonsense mutations in *FAT4* (FAT tumor suppressor, Drosophila, Homolog of, 4) in affected individuals, implicating mutations in this gene as a cause of the condition Van Maldergem syndrome 2.

*FAT-4* encodes a protein that is a member of a large family of protocadherins (OMIM #612411, 2016). *Dchs1-Fat4* signaling is required during mammalian development in multiple organs, including the brain, ear, cochlea, kidney, intestine, heart, lungs, and skeleton.

The phenotypes of *Dchs1* ^−/−^ and *Fat4* ^−/−^ single mutants and Dchs1^−/−^ and *Fat4* ^−/−^ double mutant mice are highly similar [7]. Loss of *Fat4* resulted in death at birth, while mice without DCHS1 could survive for a couple weeks at most, therefore did not reproduce. Mao et al. 2015 [7] did not report any hypothalamic-pituitary or other endocrine deficiencies in these mice.

Some pups failed to grow, remain in similar size to newborns pups. The cochlea were about 20% shorter than in the wild type cochlea; they had small cystic kidneys; the sternum was both wider and shorter and also exhibited an abnormal pattern of ossification. They show a modest increase in the width of the vertebral column but only in the lumbar and posterior thoracic region. These vertebra were also narrower along the anterior/posterior axis. The lungs were decreased in size. The intestines were also shorter than their wild type siblings, about 72% in length from the stomach to the rectum. The reduction in the size of internal organs (intestine, lung, and kidney) were not simply reflections of the decreased body size, because newborn pups did not differ significantly in overall size and other organs (e.g. skeletal, liver, heart) were not significantly smaller. The hearts were not smaller, but morphogenesis was affected; they exhibited defects in atrial septation.

Additional studies in **mice** are quite informative regarding the importance of *Dchs1 Fat4* in brain development. Cadherins play an important role in the architecture of the brain, in neural development and cortical patterning.

The regulated proliferation and differentiation of neural stem cells before the generation and migration of neurons in the cerebral cortex are central aspects of mammalian development. Periventricular neuronal heterotopia, a specific form of mislocalization of cortical neurons, can arise from neuronal progenitors that fail to negotiate aspects of these developmental processes. Capello et al., 2013 [6] show that mutations in genes encoding the receptor-ligand cadherin pair *DCHS1* and *FAT4* lead to a recessive syndrome in humans (VMS) that includes periventricular neuronal heterotopia. Reducing the expression of *Dchs1* or *Fat4* within mouse embryonic neuroepithelium increased progenitor cell numbers, reduced their differentiation into neurons, and impaired their migratory capabilities to the cortical gray matter. This resulted in the heterotopic accumulation of cells below the neuronal layers in the neocortex, reminiscent of the human phenotype, manifested as a double cortex mainly in the occipital and parietal lobes. The studies of Zakaria et al. [8], provide further knowledge of problems with neuronal migration. *Dchs1-Fat4* influence planar cell polarity (PCP), the polarization of cell structures and behaviors (direction of movement) within the plane of a tissue. PCP is essential for the generation of tissue architecture during embryogenesis and for postnatal growth and tissue repair.

Mutations in human *FAT4* and *DCHS1* cause VMS, characterized by severe neuronal abnormalities indicative of altered neuronal migration.

Studies of neuronal migration using the **murine** facial branchiomotor neurons (FBM) found that neurons, normally, undergo caudal and lateral migrations tangentially, within the plane of the neuroepithelium and finally migrate radially to form a condensed nucleus within the pia layer.

Tangential migrations contribute extensively to the architecture of the brain. *Fat4* and *Dchs1*, key components of Fat 4-PCP signaling, are expressed in complementary gradients and are required for the collective tangential migration of FBM and for their PCP. Neuronal migration in Fat^−/−^ and Dchs^−/−^ mouse mutants disrupted migration laterally, delayed migration along the rostro-caudal axis and slowed radial migration [8]. Their studies identify Fat-PCP as a novel neuronal guidance system, and are in support of the disruptions observed in migration in mice and patients as periventricular neuronal heterotopia.

The Frizzel-Flamingo (Fz-PCP) pathway plays fundamental roles in neural development, including tangential migration of FBM and olfactory neurons. Whether the disruption of lateral migration in *Fat4* ^−/−^ and *Dchs1* ^−/−^ mouse affected migration of olfactory neurons to the hypothalamus was not stated.

Bested et al. [9] addressed the question of functional cerebral asymmetries in a patient with Van Maldergem Syndrome. Mammalian cadherins *DCHS1-FAT4* affect functional cerebral architecture. Cadherins play a central role in structural left-right differentiation during brain and body development. Using neurophysiological (EEG) data, they show that when key regulators during mammalian cerebral cortical development are disrupted due to *DCHS1-FAT4* mutations, functional cerebral asymmetries are stronger. This suggests that the strength and emergence of functional cerebral asymmetries is a direct function of proliferation and differentiation of neuronal stem cells. These results support the recent assumption that the molecular mechanisms establishing early left-right differentiation are an important factor in the ontogenesis of functional lateralization.

Two additional patients with VMS were reported for surgical rehabilitation of hearing impairments [10, 11].

The molecular genetic basis for endocrine anomalies observed in some VMS patients, including ours, remains unexplained. Here, we report detailed clinical and molecular genetic studies on a female VMS patient with hypogonadotropic hypogonadism and amazia. Whole exome sequencing identified compound heterozygous pathogenic missense variants in *DCHS1*, revealing a diagnosis of VMS.

## Case presentation

Our patient, from normal parents (unaware of any consanguinity) of European ancestry, was seen when she was 13 years 2 months for short stature. The birth weight was 2.8 kg and length 53.3 cm after 9-month normal pregnancy. The Apgar scores were 3 and 8 and she had feeding problems early in life. She was evaluated by a clinical geneticist when she was 4 years of age because of dysmorphic features, but no syndrome was identified, and the chromosomes were normal, 46,XX.

### Growth & development

She had been mildly developmentally delayed since birth and had dysmorphic features: prominent forehead, epicanthal folds, hypertelorism, narrow palpebral features, neural vision impairment, maxillary hypoplasia, flat face, flat broad root of the nose, pear shaped nose, small low-set ears, flat helix, sensorineural hearing loss, wide flat palate, dental malocclusion, narrow hands and fingers, hypothenar hypoplasia, small hands and feet, cubitus valgus, genu varum, genu recurvatum, asymmetry of the legs with the left shorter than the right (2.5 cm), scoliosis and osteopenia.

**Fig. 1:**
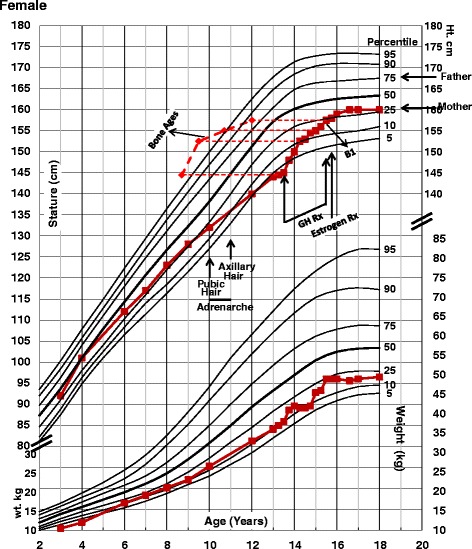
Growth chart of patient (National Center of Health Statistics 1976–1980). B1 = Tanner breast 1. GH Rx = Growth Hormone treatment

She had been growing in height along the 25th percentile, but her growth slowed down at 10 years of age. At 13 years of age she was on the 3rd percentile line (−1.88 SD). In weight, she grew for years along the 10th percentile line (Fig. 1). She had had pubic hair for 3 years and also axillary hair, but no breast development. The adrenal activity as judged by the levels of dehydroepiandrosterone sulfate (100.4 mcg/dl) was normal, within the range seen in adrenarche (20–535 mcg/dl). The bone age when she was 13 years 2 months was 9 years. Serum growth hormone (GH) after stimulation with arginine and L-dopa showed low values of 4 and 4.2 ng/ml (normal >10). Because of the short stature, lack of gonadal activity and possible, but not certain, growth hormone deficiency, she was treated with GH for 2 years from 13 ½ to 15 ½ and responded well. With the GH treatment, she went from the 3rd percentile (−1.88 SD) to the 25th percentile (−0.67 SD) and she had an adult height of 160 cm, the same as the mother (Fig. 1). Serum GH levels after stimulation tests, when she was 26 years and 7 months old were normal, 9.4 ng/ml (normal >5 ng/ml), indicating normal GH secretion.

MRI of the brain with axial and coronal images was obtained when she was 13 1/12 years. There was interference in the axial views on the frontal lobes from patient’s dental braces. No definite abnormalities were seen on the visualized portions of the brain.

### Breast aplasia/hypoplasia

At the age of 15 years and 3 months, our patient had no breast or nipple development. Serum FSH 0.3 mIU/ml, LH <0.5 mIU/ml, and estradiol 0.5 pg/ml were low (prepubertal levels). Serum FSH and LH levels after gonadotropin releasing hormone stimulation increased from <2.0 and <3.0 mIU/ml to 2.7 and 7.5 mIU/ml, a prepubertal response, consistent with idiopathic hypogonadotropic hypogonadism. She had no anosmia or hyposmia. The ultrasound of the pelvis showed small ovaries. The serum prolactin (7 ng/ml) and thyroid hormones (free T4 and TSH) were normal, as were the serum cortisol 7.8 and 14 mcg/dl (normal 3 to 21) and 17-hydroxyprogesterone <31.2 ng/dl (normal 18–220).

Because of the lack of breast development, treatment with ethinyl estradiol in small amounts, 5 mcg and later 20 mcg daily, to attain better breast growth, was begun when she was 15 ½ years. She had vaginal spotting 6 months after initiation of estradiol therapy, the nipples were developing and the breast tissue was 5 cm in width and flat (Tanner breast 2). Because of the spotting, she was given a combined contraceptive containing estrogen and progestin (ethinyl estradiol 35 mcg with ethynodiol diacetate, 1 mg) for 28 days, every month. She had menstrual periods monthly, normal development of nipples (0.8 cm diameter) and areolas (3 cm in diameter). An areolar mound was evident 3 years later, but the breasts did not grow. The maximum breast tissue detected for the next 11 years, on treatment, was again 5 cm in width and flat, with no evidence of increase in volume.

The estrogen was increased (ethinyl estradiol 50 mcg with norgestrel 0.5 ng) for 28 days every month. Menses remained normal, nipples the same, but the breasts regressed, with no breast detected on the left, and 3 cm on the right, after 1 year. At the age of 35 years, there was no breast tissue detected; menses, nipples and areolas were normal. The external genitalia were normal female. The patient has been on a contraceptive, calcium carbonate plus vitamin D (600 mg of calcium and 800 IU of vitamin D) daily, multivitamins and Alendronate 70 mg PO weekly for years.

### Skeletal dysplasia

**Fig. 2:**
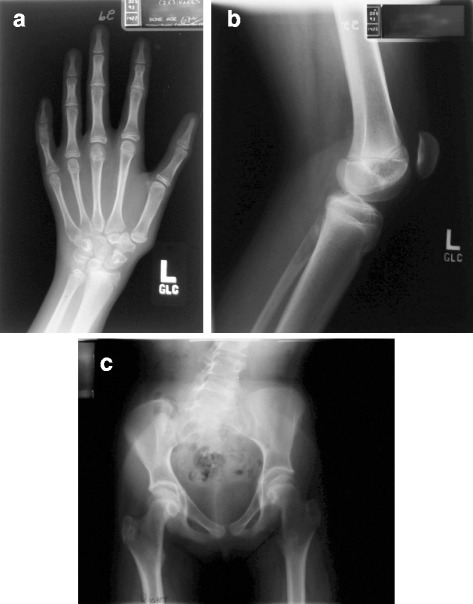
**a** Radiograph of the left hand for bone age: The distal phalanx of each digit is small and narrow. Shortening of the middle phalanx of digits 2 and 5. Slender metacarpals 4 and 5 and short 5th metacarpal. The margins of the carpal bones are more angular than smooth. **b** Lateral radiograph of the Left Knee: The patellar height ratio, measured length of the patellar tendon divided by length of the patella is 1.7, normal is 1.5. Typically increased PHR is associated with increased risk of patella dislocation. **c** AP standing pelvis radiograph: Pelvic tilt demonstrating a leg length discrepancy. The iliac bones are narrow. Bilateral Coxa Valga is present. Acetabular coverage is good. Dysplasia of the femoral epiphysis (flat and small)

The patient has also exhibited some other skeletal changes: a lumbar levoscoliosis of 40 degrees, a thoracic dextroscoliosis of 10 degrees and osteopenia. A DXA was performed when she was 30 years of age, after she had been on estrogen for 15 years and was having menses. The manufacturer’s Z-Score for the lumbar bone mineral density was −1 8, −2.3 for the right total hip, and −1.1 for total bone mineral content. No adjustment was made for the different bone volume of the femoral dysplasia. Other radiographical changes have been: dysplasia of the femoral heads, with no dislocation; abnormalities of the left hand with thin 4th and 5th metacarpals; short 5th metacarpal; short middle phalanges of the 2nd and 5th digits; unequal leg length (2.5 cm); and the margins of the carpal bones more angular than smooth (Fig. 2).

### Other

Other test results throughout the years were normal and include: thyroid hormones (free T4 and TSH), adrenal hormones, metabolic screening, electrolytes, cholesterol, liver tests, BUN and creatinine, calcium, alkaline phosphatase, CBC, IGF-1 and vitamin D-25-hydroxy. Urinary calcium creatinine ratio has been normal.

At the age of 37 years, the patient’s height was 160 cm (25th percentile) and weight was 54.1 kg (40th percentile). The blood pressure was normal. The dysmorphic features noted previously were present. Otherwise, the findings were normal in the neck, thyroid gland, heart, lungs and abdomen. The scoliosis remained. The pubic and the axillary hair were normal for an adult female and nipples were well developed, measuring 0.8 cm in width. Breast tissue could not be felt on the left or on the right.

### Genetic evaluation

#### Genetic methods

Our molecular genetic studies were prospectively reviewed and approved by the Institutional Review Board (IRB) of The Research Institute at Nationwide Children’s Hospital. Consent was obtained from the patient and parents for whole exome sequencing (WES). All genomic DNA samples in the study were processed using Agilent SureSelectQXT Target Enrichment System for Illumina Paired End Sequencing Protocol (Agilent Technologies, CA). Genomic DNA was sheared and target exonic regions were captured with the Agilent SureSelect Clinical Research Exome kit. Paired-end 151 base pair reads were generated for exome-enriched libraries sequenced on the Illumina HiSeq 4000 (Illumina, CA). All samples were sequenced to a depth of 100X.

Secondary analysis was performed using Churchill, a pipeline developed in house for the discovery of human genetic variation [12]. Churchill utilizes the Burrows-Wheeler Aligner (BWA) to align sequence data to the reference genome (build GRCh37). Further refinement steps were performed on the aligned sequence data following the Broad Institutes guidelines for best practices (https://www.broadinstitute.org/gatk/guide/best-practices). Duplicate sequence reads were removed using PicardTools (v1.117). Local realignment was performed on the aligned sequence data using the Genome Analysis Toolkit (v3.4–0). Churchill’s own deterministic implementation of base quality score recalibration was used. The GATK’s HaplotypeCaller (HC) was used to call variants. To maximize sensitivity, variant calling was performed across all samples in the study. Use of the GATK’s variant quality score recalibration (VQSR) was excluded in favor of using Churchill’s own quality-based variant filtering algorithm. Comparison of variant calling performance with the NIST Genome in a Bottle (GIAB) gold reference standard (version 3.22) utilizing whole genome sequencing data for NA12878 (30X) demonstrated that Churchill is 99.9985% accurate, with a sensitivity (recall) of 99.6061%.

Tertiary analysis was performed using ANNOVAR and custom in-house scripts to annotate the variant call set with mutation and gene information, protein functional predictions, and population allele frequencies [13]. Common variation occurring at greater than 1% mean allele frequency (MAF) in the population was excluded, and variants outside of coding regions (defined as greater than 4 base pairs from an exon splice site) as well as exonic variants coding for synonymous single nucleotide polymorphisms were also dropped. Variants were further filtered based on the pattern of inheritance expected from examination of the pedigree.

#### Genetic results

Previously, karyotypes from the blood and the skin were normal (46, XX). A microarray comparative genomic hybridization (aCGH) showed an approximate 62.1 kb gain within chromosome 13, band q34. This region does not contain any genes with the UCSC genome browser (genome.ucsc.edu) and is in a region with limited copy number variation [14, 15]. Consequently, the laboratory report indicated that this finding is likely a benign copy number variation.

**Fig. 3:**
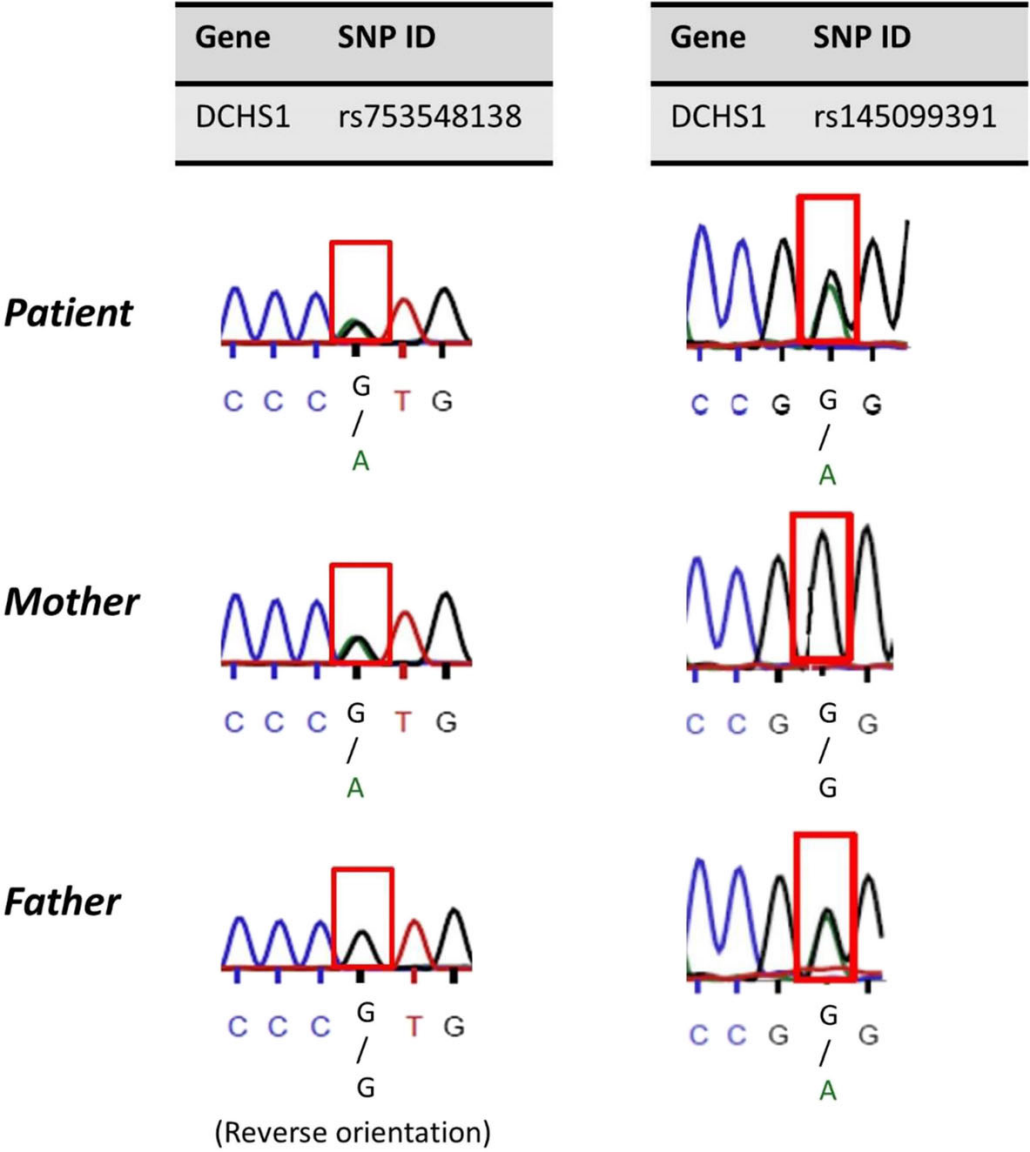
Validation of NGS sequencing by Sanger Sequencing: Sequence chromatograms show the compound heterozygous mutation detected in the patient in the *DCHS1* gene in exon 19 (left) and exon 2 (right). The patient’s mother and father were also re-sequenced to verify transmission of one mutation from each parent. *DCHS1*: RefSeq NM_003737.3

Clinically significant results from whole exome sequencing were validated by Sanger dideoxy sequencing of targeted DNA fragments, confirming that the patient had compound heterozygous pathogenic variants (rs145099391;P197L & rs753548138;T2334 M) in the *DCHS1* gene (RefSeq NM_003737.3) (Table 2). Each parent was confirmed to be a heterozgyous carrier of one of the mutations (Fig. 3). Both of these *DCHS1* variants have been reported in a heterozygous state in individuals of European descent, albeit very rarely (Table 2), but neither of them have been observed in a homozygous state in an individual, according to the genome Aggregation Database (gnomAD) of >15,000 healthy individuals of European ancestry [16]. We did not identify any known pathogenic variants in our patient in genes that have been reported to be associated with hypogonadotropic hypogonadism [17].

**Table 2:** Summary of *DCHS1* Variants Detected in Patient

Chr	Gene	Position^a^	dbSNP ID	SNP	Residue change	European population frequency (gnomAD database)	Type	Protein prediction CADD^b^ Score
11p15.4	*DCHS1*	6,662,255	rs145099391	G > A	p.P197L	8.89 × 10^−4^	Missense Exon 2	22.30
11p15.4	*DCHS1*	6,646,574	rs753548138	G > A	p.T2334 M	9.02 × 10^−6^	Missense Exon 19	23.30

^a^RefSeq NM_003737.3

^b^CADD scores are derived from several different functional annotation tools. A score of 20 means that a variant is amongst the top 1% of deleterious variants in the human genome. The higher the score, the more likely that variant is predicted to be deleterious to the protein [33]

## Discussion

Our patient did not have growth hormone deficiency. The GH level after stimulation tests as an adult was normal indicating normal GH secretion [18, 19]. The low levels of GH after stimulation tests, at prepubertal age, of <10 ng/ml do not certainly indicate GH deficiency. Overlap exists in peak GH concentrations between normal children and those with GH deficiency. As many 30% of normal children may have peak values of <10 ng/ml in provocative tests [20]. There was no evidence of other pituitary deficiencies (prolactin, TSH, ACTH). The slow growth beginning at 10 years of age, lack of pubertal spurt, delayed bone age, and osteopenia are consistent with estrogen deficiency. She is clearly affected with Van Maldergem syndrome 1, sharing the clinical features described in previously reported patients and confirmed by our finding of compound heterozygous pathogenic variants in *DCHS1*. She also had idiopathic hypogonadotropic hypogonadism and after estrogen treatment, amazia (aplasia/hypoplasia of the breast, but normal nipples and areolas). Amastia is characterized by no breasts and no nipples, and athelia, by normal breasts and no nipples, terminology used to describe congenital abnormalities of the breasts (OMIM %113,700).

No endocrine abnormalities were evaluated in previously published patients with VMS 1 or 2 (Table 1). However, by the changes in two VMS patients described previously, some may have been affected also by hypogonadotropic hypogonadism. Six of the 13 patients reported previously were too young to make such conclusions, 4 to 9 years of age. In a 9-year-old female and a 14-year-old male there was no mention of the pubertal changes. A follow up of the original 11 year-old female patient of Van Maldergem, at the age of 32 years did not have any breast or pubertal development [4] suggesting the possibility of hypogonadotropic hypogonadism, and, with no adrenarche, possibly ACTH deficiency. Another patient [6] had microphallus, bifid scrotum, and bilateral cryptorchidism at birth, which are findings that may be present in patients with congenital hypogonadotropic hypogonadism. Hypoplasia of the mammillae was also a finding. Whether eleven of the previously reported patients also had hypogonadotropic hypogonadism and breast and/or nipple aplasia/hypoplasia is unknown. Present evaluation of the patients previously reported would be helpful.

### Hypogonadotropic Hypogonadism

The cause of hypogonadotropic hypogonadism is consistent with congenital gonadotropin releasing hormone (GnRH) deficiency, owing to the functional absence of GnRH secretion, from hypothalamic hypophysiotropic neurons associated with congenital malformations with craniofacial anomalies or GnRH receptor deficiency with absence of gonadotropin secretion. No anosmia or hyposmia was present in our patient (or were reported in any of the patients) to suggest Kallmann syndrome. There were no findings to suggest an acquired cause, such as hypothalamic tumors, head trauma, hypothyroidism, or systemic diseases.

The presence of normal adrenarche, in the absence of breast development, practically excludes constitutional delay of puberty, where pubic hair is typically absent [21, 22]. Inactivating mutations for the genes encoding idiopathic hypogonadotropic hypogonadism [17] were not identified.

### Breast development and regulation

The breasts are composed of epithelial branching ducts which connect the functional units of the breast (the lobules) to the nipple and the stroma containing fat and connective tissue. The stroma comprises the majority of the breast volume in the non-lactating state [23].

Estrogen (estradiol, E_2_) is the major factor in promoting breast development by activating the estrogen receptor α (ESR1) but there are different pathways; the classic genomic pathway and alternatives.

In the classic pathway, the activated ESR1 dimerizes, binds with high affinity and specificity to DNA sequences called estrogen response elements (EREs) to regulate transcription rates of target genes (Additional file 1: Figure S1A).

There are alternative mechanisms of action. Estrogen receptors can modulate activation of reporter genes containing activating protein (Ap-1) elements (Additional file 1: Figure S1B). Ap-1 is a transcription factor complex that interacts with Ap-1 sites in gene promoters to activate a large number of genes involved in cellular differentiation and development; Ap-1 may be important for E_2_ dependent activation or repression of the progesterone receptor, gonadotropin releasing hormone receptor, prolactin, and other genes [24].

### Breasts and/or nipples aplasia or hypoplasia

Normal mammary growth differentiation and regression is the result of complex interactions between systemic hormones (estrogens and progesterone) and local cell interactions which are mediated by a variety of growth factors, including epidermal growth factor, transforming growth factor, and fibroblast growth factor [22]. Because of this complexity, the cause of the breast and/or nipple aplasia or hypoplasia is rather complex and difficult to establish.

Aplasia or hypoplasia of the breasts and/or nipples are rare anomalies. These rare anomalies have been reported in 2 isolated patients and in several generations in 3 familial patients (OMIM %113,700), but the genetic cause was not determined. Mammary hypoplasia is quite frequent in Meier-Gorlin syndrome, a disorder resulting from 5 different genes from the pre-replication complex. All 13 post-pubertal females (100%) had breast hypoplasia [25]. No genetic cause was established for the breast hypoplasia. Amastia (characterized by no breasts and no nipples) or severe unilateral hypoplasia is associated with hypoplasia of the pectoral muscle in 90% of the patients with Poland syndrome, which is characterized by hypoplasia or absence of the pectoral muscle and abnormalities of the ipsilateral arm, thought to be due to abnormal perfusion from the axillary artery. Absence of the breasts could occur in ectodermal dysplasia. The author saw a family, many years ago, with ectodermal dysplasia. The girl, 16 years of age, her mother, and maternal aunt had normal breast and nipples on the right and no breast or nipples on the left. It is known that congenital ectodermal defects are associated with amastia and affect male and female patients. Because it is a sex-linked trait, males present with more severe malformation. The typical patient presentation includes bilateral amazia or amastia with associated abnormalities of the skin and appendages (hair, eccrine glands, and sebaceous glands), teeth and nails [26, 27].

The gene for X-linked anhidrotic ectodermal dysplasia is EDA on Xq.31 (OMIM, 305,100).

The only genetic abnormalities reported in patients with aplasia or hypoplasia of the breasts or nipples were the following:

1. An 18 years old female with a homozygous mutation on the ligand domain of the ESR1, gene, rendering the ESR1 functionally inactive, who had amastia and infertility [28].
2. Two siblings and a cousin who had unilateral or bilateral athelia with a homozygous mutation in the *PTPRF* gene (protein tyrosine phosphatase receptor type F) in chromosome 1p32.2 [29]. The *PTPRF* gene (also called the Leukocyte Antigen-Related Tyrosine phosphatase or *LAR* gene) encodes a membrane protein. Mammary glands of homozygous *Lar* deficient female mice were incapable of delivering milk due to an impaired terminal differentiation of alveoli in late pregnancy [30].

Whole exome sequence analysis of our patient revealed that both ESR1 and PTPRF contained no potentially pathogenic variants.

### Causative factors of breast aplasia/hypoplasia and/or hypogonadotropic hypogonadism

The lack of breast development in our patient, at the beginning, was clearly the result from lack of estrogen due to hypogonadotropic hypogonadism. With estrogen treatment, there was a response with development of the ducts and nipples. The cause of the hypoplasia of breasts, mainly consistent with an impaired development of the stroma, and particularly, the rapid and early regression of breast stroma, when taking a combined estrogen/progestin daily, is more difficult to know. This is particularly difficult, given the complexity of the regulation of breast development, and the different pathways, many of them based mainly on observations obtained from mice and cells.

Possibilities could be related to repressive or antagonistic factors of **activator protein 1 (Ap-1)**:

- The repression of GnRH receptor expression by Ap-1. It is recognized that estrogen is one of the most important regulators of GnRH receptor and represses the expression of human GnRH receptor via ESR1 and an Ap-1 motif. Ap-1 could repress the expression of GnRH receptor and be the cause of congenital hypogonadotropic hypogonadism [24]. This could occur during embryogenesis. Estrogen and Ap-1 have biological effects on the neurons, hypothalamus and pituitary [24].
- Ap-1 could also repress the expression of the progesterone receptor [31]. This could explain the impaired development of the breast stroma, and the rapid breast regression when ethinyl estradiol treatment was increased from 35 to 50 mcg, possibly activating Ap-1.
- Sometimes, in the presence of E_2_, ESR2 can oppose the agonist action of Ap-1 reporter genes [32]. When the two ER subtypes are co-expressed in cells, ESR2 can antagonize ESR1 dependent transcription. It was shown that for ESR1 mediated regulation of Ap-1 dependent transcription, ESR2 expression alters the recruitment patterns of c-Fos to Ap-1 regulated promoters.

### Exome sequence interpretation

VMS-1 is caused by recessive mutations in the *DCHS1* gene. In our patient, we identified two rare, missense variants in *DCHS1* in a compound heterozygous state. Interestingly, neither of the variants detected in our patient have been observed in a homozygous state in an individual, supporting our conclusion that these two variants together in our patient can be disease-causing for the documented syndrome VMS-1, an autosomal recessive disorder. Both *DCHS1* variants were predicted to be likely damaging to protein according to CADD scores (Table 2) [33].

In addition to the *DCHS1* variants, we identified a de novo coding variant (c.1643G > A; p.R548Q; RefSeq NM_138477) in *CDAN1* gene of unknown significance. The R548Q variant was not reported in gnomAD, indicating it is not a common benign variant in European populations. The R548Q variant is a “semi-conservative” amino acid substitution, as these amino acid residues (arginine (R) and glutamine (Q)) share some common properties (e.g., polarity and size) but differ in charge and some other properties. This substitution occurs at a position that is highly conserved across several species. In silico analysis consistently predicts this variant is damaging to the protein structure and/or function. The only reported phenotype associated with *CDAN1* variation in humans is congenital dyserythropoietic anemia type I, characterized by recessive or compound heterozygous mutations in *CDAN1* [34]; there are no reported disease associations with *CDAN1* heterozygous variants. This gene encodes a protein that appears to play a role in nuclear envelope integrity, possibly related to microtubule attachments. Based on the lack of literature on heterozygous CDAN1 variants in human disease, we cannot conclude if the R548Q variant is related to the patient’s phenotype, or just a rare, benign, de novo variant. We do, however, feel it necessary to report this finding in our manuscript so that other readers may be fully aware of our exome findings.

## Conclusion

This patient highlights an individual with VMS, characterized by compound heterozygous variants in *DCHS1*, a rare syndrome reported in only 13 patients, based on our literature search. Our observations provide additional information on the phenotypic spectrum of VMS, including hypogonadotropic hypogonadism and amazia (aplasia/hypoplasia of the breasts but normal nipples and areolas), that was not previously reported with VMS. Congenital breast aplasia/hypoplasia, in the presence of estrogen and progesterone is a rare anomaly of breast development, which pathogenesis is not well established.

## Additional file

Additional file 1: Figure S1A. Pathways of estrogen activation of gene transcription (Classic): E_2_ = Estradiol, ER = Estrogen Receptor, ERE = Estrogen Receptor Elements. Estrogen (estradiol, E2) is the major factor in promoting breast development by activating the estrogen receptor α (ESR1) but there are different pathways; the classic genomic pathway and alternatives. In the classic pathway (Figure S1, A), the activated ESR1 dimerizes, binds with high affinity and specificity to DNA sequences called estrogen response elements (EREs) to regulate transcription rates of target genes. Activated ESR1 then recruits-interacts with steroid receptor co-regulators (SRCS) and chromatin remodelers that facilitate access to chromatin and coordinate transcription of the transcriptional modulators. Transcription is facilitated or impeded in part by modifications of histones, the more abundant proteins in the nucleus which package the DNA. Modification of histones by acetylation via histone acetyl transferases or deacetylation via histone deacetylases affects transcription of genes [22]. **Figure S1B.** There are alternative mechanisms of action when the estrogen receptor can sometimes regulate expression of genes that lack EREs resulting in activation of reporter genes containing activator protein 1 (Ap-1) elements (Figure S1, B). Through protein-protein interaction, estrogen receptors can modulate the transcriptional activity of heterodimers of the transcription factors fos and jun (Ap-1 responsive elements), leading to activation of reporter genes containing Ap-1 elements. This non-classical genomic pathway is also functional in vivo. Ap-1 is a transcription factor complex containing the proto-oncogenes jun/fos and other family members. This complex interacts with Ap-1 sites in gene promoters to activate a large number of genes involved in cellular differentiation and development [16]. (DOCX 96 kb)

## Abbreviations

aCGH: Microarray-based Comparative Genomic Hybridization
ACTH: Adrenocorticotropic Hormone
AP-1: Activating Protein-1
CBC: Complete Blood Count
*DCHS1*: Dachsous Cadherin-Related 1 gene
DXA: Dual-Energy X-ray Absorptiometry (bone densitometry)
*FAT4*: FAT Atypical Cadherin 4 gene
FSH: Follicle-Stimulation Hormone
IQ: Intelligence Quotient
IRB: Institutional Review Board
LH: Luteinizing Hormone
OMIM: Online Mendelian Inheritance in Man
SD: Standard Deviation
T4: Thyroxine
TSH: Thyroid-Stimulation Hormone
VMS: Van Maldergem Syndrome
WES: Whole Exome Sequencing
