# Supplementary material for: A patient with van Maldergem syndrome with endocrine abnormalities, hypogonadotropic hypogonadism, and breast aplasia/hypoplasia
Source: Int J Pediatr Endocrinol. 2017 Oct 13;2017:12. doi: 10.1186/s13633-017-0052-z (PMC5640965; doi:10.1186/s13633-017-0052-z)
Supplement: Additional file 1: Figure S1A. — Pathways of estrogen activation of gene transcription (Classic): E2 = Estradiol, ER = Estrogen Receptor, ERE = Estrogen Receptor Elements. Estrogen (estradiol, E2) is the major factor in promoting breast development by activating the estrogen receptor α (ESR1) but there are different pathways; the classic genomic pathway and alternatives. In the classic pathway (Figure S1, A), the activated ESR1 dimerizes, binds with high affinity and specificity to DNA sequences called estrogen response elements (EREs) to regulate transcription rates of target genes. Activated ESR1 then recruits-interacts with steroid receptor co-regulators (SRCS) and chromatin remodelers that facilitate access to chromatin and coordinate transcription of the transcriptional modulators. Transcription is facilitated or impeded in part by modifications of histones, the more abundant proteins in the nucleus which package the DNA. Modification of histones by acetylation via histone acetyl transferases or deacetylation via histone deacetylases affects transcription of genes [22]. Figure S1B. There are alternative mechanisms of action when the estrogen receptor can sometimes regulate expression of genes that lack EREs resulting in activation of reporter genes containing activator protein 1 (Ap-1) elements (Figure S1, B). Through protein-protein interaction, estrogen receptors can modulate the transcriptional activity of heterodimers of the transcription factors fos and jun (Ap-1 responsive elements), leading to activation of reporter genes containing Ap-1 elements. This non-classical genomic pathway is also functional in vivo. Ap-1 is a transcription factor complex containing the proto-oncogenes jun/fos and other family members. This complex interacts with Ap-1 sites in gene promoters to activate a large number of genes involved in cellular differentiation and development [16]. (DOCX 96 kb) [file 13633_2017_52_MOESM1_ESM.docx]

# Additional file 1

**Figure S1A**

**Classic Pathway**

**DNA Binding**

ER

ER

E_2_

E_2_

EREs

E_2_

ER

Nuclear

Membrane

Coactivators

Chromatin

remodelers

RNA transcription

of genes

DNA

ER

ER

E_2_

E_2_

E_2_

ER

Membrane

Transcription Factors Complexes

DNA Activation of genes

**Alternative Pathway**

**DNA Binding Independent**

**Protein-protein interaction and no EREs**

**Activation of reporter genes containing Ap-1 elements**

fox jun Ap-1 responsive elements

**Figure S1B**
